# Single-Molecule Surface-Enhanced Raman Spectroscopy

**DOI:** 10.3390/s22134889

**Published:** 2022-06-29

**Authors:** Yuxuan Qiu, Cuifang Kuang, Xu Liu, Longhua Tang

**Affiliations:** 1 State Key Laboratory of Modern Optical Instrumentation, College of Optical Science & Engineering, Zhejiang University, Hangzhou 310027, China; yuxuanqiu@zju.edu.cn (Y.Q.); cfkuang@zju.edu.cn (C.K.); liuxu@zju.edu.cn (X.L.); 2 Ningbo Research Institute, Zhejiang University, Ningbo 315100, China; 3 Collaborative Innovation Center of Extreme Optics, Shanxi University, Taiyuan 030006, China

**Keywords:** surface-enhanced Raman spectroscopy, single-molecule detection, surface plasmon resonance, nanoparticle

## Abstract

Single-molecule surface-enhanced Raman spectroscopy (SM-SERS) has the potential to detect single molecules in a non-invasive, label-free manner with high-throughput. SM-SERS can detect chemical information of single molecules without statistical averaging and has wide application in chemical analysis, nanoelectronics, biochemical sensing, etc. Recently, a series of unprecedented advances have been realized in science and application by SM-SERS, which has attracted the interest of various fields. In this review, we first elucidate the key concepts of SM-SERS, including enhancement factor (EF), spectral fluctuation, and experimental evidence of single-molecule events. Next, we systematically discuss advanced implementations of SM-SERS, including substrates with ultra-high EF and reproducibility, strategies to improve the probability of molecules being localized in hotspots, and nonmetallic and hybrid substrates. Then, several examples for the application of SM-SERS are proposed, including catalysis, nanoelectronics, and sensing. Finally, we summarize the challenges and future of SM-SERS. We hope this literature review will inspire the interest of researchers in more fields.

## 1. Introduction

Raman scattering arises from the interaction between photons and molecules [1]. The molecular transition energy can be obtained by measuring the wavelength of Raman scattered photons. Thus, the Raman spectrum is also known as the chemical fingerprint spectrum [2]. Raman spectroscopy has the advantage of being label-free and non-invasive, so it is widely used in material and life science. However, spontaneous Raman scattering is very weak, with only 1 in 10^7^ photons involved, which limits the sensitivity of Raman spectroscopy.

Surface-enhanced Raman spectroscopy (SERS) significantly enhances the Raman signal beyond the sensitivity limit of Raman spectroscopy [3]. In general, surface-enhanced Raman scattering occurs on molecules adsorbed on the rough surface or nanostructure of noble metal substrates. It is generally accepted that SERS enhancement is the result of electromagnetic mechanisms (EM) and chemical mechanisms (CM) [4,5]. EM enhancement is the dominant contribution of SERS. Surface plasmon resonance (SPR), the collective oscillation of free electrons excited by photons, localizes the electromagnetic field on the surface of the substrate. The strong, localized electromagnetic field amplifies the Raman signal of molecules adsorbed on the substrate. CM, providing less enhancement than EM, is employed as a complementary approach. The Raman polarizability tensor of analyte molecules is modified by charge transfer between molecules and hot electrons.

**Figure 1:**
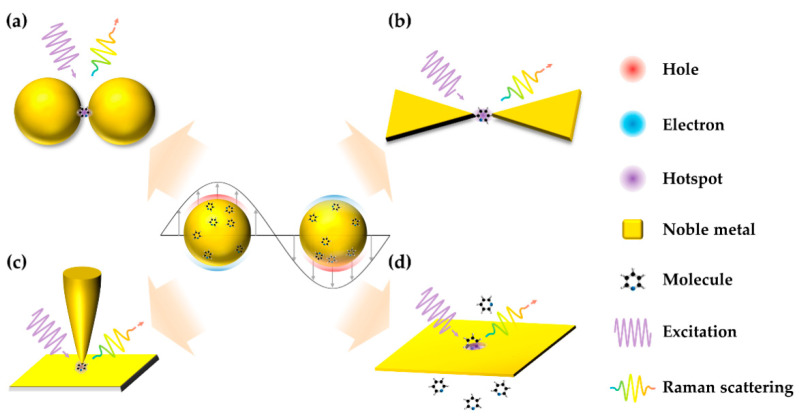
Schematic diagram of SM-SERS. Center: SPR effect of nanoparticles; Inset: (**a**) dimetric nanostructure; (**b**) plasmonic single-molecule junction; (**c**) plasmonic tip; (**d**) plasmonic nanopore.

Single-molecule SERS (SM-SERS), the Raman spectrum from a single molecule, is the ultimate goal of SERS in terms of sensitivity (Figure 1). SM-SERS is an unprecedentedly high-throughput and ultra-sensitive method for chemical analysis. More importantly, subtle spectroscopic phenomena from single molecules are observed without a statistical average. In application, SM-SERS has been widely used in various fields, including chemical analysis, nanoelectronics, and biochemical sensing [6,7,8,9]. For example, in situ monitoring of catalytic reactions could be achieved by SM-SERS. Monitoring at the single-molecule level reveals the mechanism of the reaction, thus guiding the design of highly efficient catalysts.

It has been more than 20 years since SM-SERS was first reported in 1997 [10,11]. In these two decades, the theory of SM-SERS has undergone several changes, but some experimental phenomena have yet to be fully explained. A series of sophisticated schemes for SM-SERS has been proposed, with a strong localized electromagnetic field, high-reproducibility substrates, strategies for localizing molecules in hotspots, etc. Researchers are also extending SM-SERS to a variety of environments to accommodate more applications. In practice, some critical challenges still exist. In this review, we clarify the concepts of SM-SERS, evaluate the progress and challenges in SM-SERS, and illustrate applications of SM-SERS. We hope this literature review will inspire the interest of researchers in various fields.

## 2. Single-Molecule SERS

Measuring the structure and behavior of single molecules has attracted great attention in various fields. Several single-molecule detection techniques have been implemented [12,13], including transmission electron microscopy (TEM), scanning tunneling microscopy (STM), atomic force microscopy (AFM), tunneling sensing, single-molecule fluorescence microscopy, etc. SM-SERS detects the vibrational modes of single molecules, providing high-throughput structural information. SM-SERS could be flexibly applied in various environments, from ultra-high vacuum at cryogenic temperature to in a solution at room temperature [14,15]. In addition, SM-SERS plays an important role in catalysis, nanoelectronics, sensing, etc. SM-SERS has demonstrated exciting prospects in science and application. However, when the concentration of the analyte is decreased to a single-molecule level, extraordinary phenomena and problems appear. Several concepts of SM-SERS are worth clarifying.

### 2.1. Enhancement Factor

The enhancement factor (EF) of SERS is the ratio of Raman signal with and without substrates in the same measurement conditions, normalized for the number of molecules probed. The EF is defined as:

(1) $$\mathrm{EF}=\frac{I_{SERS}/N_{surf}}{I_{RS}/N_{vol}}\;,$$

where $I_{RS}$ and $I_{SERS}$ are the Raman intensity for $N_{vol}$ molecules and SERS intensity for $N_{surf}$ molecules.

EM-induced enhancement comes from two multiplicative effects: one results from enhancement of the local excitation field; the other is due to enhancement of the re-emitted Raman scattering [16].

(2) $$G(r)={\left[\frac{E_{loc}(\omega_{0},r)}{E_{0}(\omega_{0},r)}\right]}^{2}{\left[\frac{E_{loc}(\omega_{R},r)}{E_{0}(\omega_{R},r)}\right]}^{2}\;,$$

where r is the spatial coordinate, $\omega_{0}$ and $\omega_{R}$ are the frequency of excitation and Raman photons, $E_{0}\left(\omega ,r\right)$ is the electric field of excitation light, and $E_{loc}\left(\omega ,r\right)$ is the enhanced local electric field. Ignoring the frequency difference between the excitation and Raman photons, EM-induced enhancement is approximately proportional to the fourth power of the local electric field enhancement, which is known as the E^4^ approximation [17].

CM-induced enhancement relies on modification of the Raman polarizability tensor of the analyte molecules by charge transfer. The overall SERS intensity $I\left(\omega_{R}\right)$ can be expressed as:

(3) $$I(\omega_{R})=AG(r){\left|\alpha (\omega_{R},\omega_{0})\right|}^{2}I_{0}(r,\omega_{0})$$

where A is the collection efficiency of the setups, $\alpha \left(\omega_{R},\omega_{0}\right)$ is the Raman polarizability of the molecule, and $I_{0}\left(r,\omega_{0}\right)$ is the intensity of excitation light.

The EF required to observe single-molecule events is critical but controversial. When SM-SERS was first observed, single-molecule events were observed when the molecules were adsorbed in the Ag colloid. It was claimed that an EF of 10^14^ is essential for single-molecule detection [11]. A series of subsequent studies revised the value to 10^7^–10^8^ [18,19].

Indeed, SERS intensity depends not only on the SERS substrates, it is also related to the analytes and optical setups. For analytes, various molecules differ in Raman scattering cross sections. In the same excitation conditions, the larger the differential cross sections, the more intense the Raman scattering [20]. Especially when the excitation wavelength corresponds to the electronic transition of a molecule, resonance enhancement of Raman scattering occurs. The excited electrons transition to the excited electronic state rather than a virtual state, significantly increasing the cross section, providing the EF of 10^2^–10^6^ [21]. As for optical setups [22], a high numerical aperture (NA) objective enables high Raman collection efficiency. The sensitivity of the spectrometer determines the lowest detectable intensity coupled to the spectrometer. In particular, when the wavelength and polarization of the excitation beam match the SPR of the SERS substrates, the highest EM-induced EF is obtained.

It is worth mentioning that the mechanism of some experimental phenomena of SM-SERS has not yet been fully explained. Several reports have proposed that some previously ignored physical mechanisms, such as Rayleigh scattering, discrete interaction, and quantum interaction need to be considered in SM-SERS [23,24,25,26].

### 2.2. Experimental Evidence of Single-Molecule Event

In earlier studies, the ultra-low concentration of the analyte was considered evidence for single-molecule detection, with less than one molecule in the probed volume on average [10]. However, due to the sparse distribution of hotspots, it is highly probable that no molecules are present in the hotspot in most events. Moreover, contamination, dilution errors, wall adsorption, and overestimation of hotspots affect the demonstration in such a low concentration. Consequently, direct experimental evidence is required to prove the existence of a single-molecule event.

Fluctuations of SERS both in intensity and spectral shape were once considered the experimental evidence of single-molecule events [10,11]. In single-molecule level concentration, the dynamic movement of molecules in hotspots causes signal fluctuations. Nevertheless, later studies showed that the fluctuations are an intrinsic property of SERS rather than adequate evidence for SM-SERS [27]. Similar fluctuations were observed at higher concentrations [28]. More evidence was considered based on statistics. When the average number of molecules in the probed volume is one or less, the statistical distribution of the Raman signal changes from Gaussian to Poisson and is quantized to correspond to zero, one and two molecules in the probed volume [10,11]. It has been noted that inadequate sampling (e.g., 100) following a log-normal distribution often exhibits oscillations similar to a Poisson distribution [27]. More than 10^4^ samplings are required for a rigorous argument [29].

Bianalyte’s approach [30] provides direct experimental evidence for SM-SERS without relying on ultra-low concentration. In this approach, a mixture of two analytes with distinguishable Raman spectra are adopted for SERS measurement. With more than one molecule in the hotspot, the SERS is a mixture spectrum of both analytes. If only one molecule on average exists in the hotspot, the measured SERS signal comes from either one of the two analytes.

### 2.3. Spectral Fluctuation and Data Analysis

To retrieve chemical information from recorded Raman spectrum, many classification and dimensionality reduction algorithms are adopted to deal with SERS data, including support vector machine (SVM), principal component analysis (PCA), cluster analysis, and deep learning [31,32,33]. However, spectral fluctuations in SM-SERS inevitably disturbs data analysis, even though some digital signal processing methods are used to suppress the effects of fluctuation [27].

In contrast, spectral fluctuations and blinking imply single-molecule information. Lindquist et al. [34] proposed high-speed super-resolution imaging to capture the temporal and spatial features of fluctuation. The physical and chemical properties of molecules and substrates, including molecular adsorption and desorption, molecular reorientation, surface diffusion, and metal surface reconstruction [35,36], have been resolved through spectral fluctuation and blinking. Weber et al. [37] mapped the shape of hotspots from the fluctuation and studied the molecular motion of the nanoparticles. Furthermore, environmental factors such as temperature, pressure, light intensity, and charge transfer have been proven to influence spectral fluctuations [38,39,40,41]. So far, the principle of spectral fluctuation and blinking has not been fully understood. Therefore, the study of spectral fluctuation is not only conducive to the understanding of the single-molecule system, but also to the development of an analysis algorithm based on SM-SERS data.

## 3. Advanced Implementation of SM-SERS

According to the reports by Kneipp and Nie [10,11], SM-SERS measurements rely on the random adsorption of analyte molecules to the hotspots in colloidal silver solution. A series of surveys revealed that only the aggregation of multiple nanoparticles could provide enough EF for SM-SERS [28,42]. When the analyte molecule is localized at the gap between the nanoparticles, the Raman scattering is greatly amplified. Although single-molecule sensitivity has been realized in such a scheme, some drawbacks still exist. First, EF is highly sensitive to interparticle distance, which cannot be precisely controlled in colloidal silver. Second, the aggregation of nanoparticles is extremely heterogeneous, and few SM-SERS hotspots are generated, which makes analyte molecules in hotspots a rare event. Therefore, an advanced SM-SERS scheme has been developed around two aspects: SERS substrate with ultra-high EF and reproducibility, and improving the probability of analyte molecules in hotspots. On the other hand, conventional SERS substrate material is dominated by a noble metal. The development of nonmetallic and hybrid substrates is beneficial to broaden the application of SM-SERS.

### 3.1. Substrates with Ultra-High EF and Reproducibility

Single nanoparticles typically do not provide enough EF for SM-SERS, and only the gaps between nanoparticles are active sites. The nanoscale error of gaps causes an EF difference up to several orders of magnitude. Nevertheless, such sites are sparse in colloidal silver. Less than 5% of sites contribute more than 80% of the overall SERS intensity [43]. Thus, constructing highly reproducible substrates is crucial for SM-SERS.

**Figure 2:**
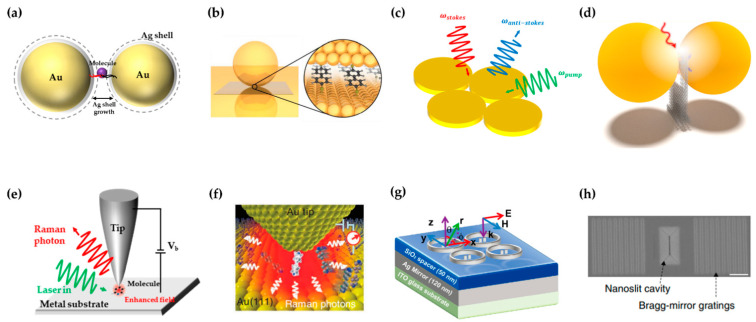
High-reproducibility substrates for SM-SERS. (**a**) Nanogap-engineered SERS-active GSND. (**b**) NPoM configuration. Figure reproduced from [46]. (**c**) The enhancement map of the quadrumer substrate. (**d**) Nanoparticle dimer assembled on the DNA origami nanofork. Figure reproduced from [57]. (**e**) Tunneling-controlled TERS. (**f**) Schematic diagram of FM-TERS. Figure reproduced from [64]. (**g**) Nanoantenna chip for SM-SERS. Figure reproduced from [66]. (**h**) The SEM image of the nanoslit device. Scale bar, 1 μm. Scanning electron microscopy, SEM. Figure reproduced from [15].

Dimer, as a simple and effective nanostructure, is widely adopted in SM-SERS due to its ultra-high EF in the nanogap. Lim et al. [44] reported a reproducible dimetric nanostructure, called gold–silver core–shell nanodumbbell (GSND) (Figure 2a). GSND consists of two Au nanoparticles with an Ag shell linked by DNA strands. A dye molecule attached to the DNA strand is located in the gap between nanoparticles. The size of the gap is precisely controlled on the nanometer scale by tuning the Ag shell thickness. The EF of GSND with a 5 nm Ag shell is 2.7 × 10^12^ and ensures a yield of 73%. Li et al. [45] achieved SM-SERS with a nanoparticle-on-mirror (NPoM) (Figure 2b) [46,47] configuration, in which the gap between the nanoparticle and the metallic surface is determined by the length of the adsorbed molecules. The configuration could detect a distinguishable signal in 10 nM and achieve single-molecule sensitivity as proven by the bianalyte approach. Further, Carnegie et al. [48] observed the optically induced formation of picocavities in NPoM, which further focused the local field down to the subnanometer scale. The picocavities were stabilized at room temperature by chemically modifying the self-assembled monolayers. When SERS is combined with nonlinear optical processes, such as stimulated Raman spectroscopy (SRS) and coherent anti-Stokes Raman scattering (CARS), more complex nanostructures are utilized [49]. Zhang et al. [50] demonstrated a Fano-resonant quadrumer substrate (Figure 2c) to achieve single-molecule surface-enhanced CARS. Pump light, Stokes scattering, and anti-Stokes scattering are simultaneously enhanced with the quadrumer substrate. An EF of 10^11^ over spontaneous Raman scattering was realized, which is capable of single-molecule detection.

DNA origami [51], a powerful nanoengineering tool, offers an elegant approach to designing sophisticated nanostructures. In DNA origami, a long scaffold single strand and a set of short artificial staple strands are self-assembled in various 2D and 3D structures. Thacker et al. [52] assembled two 40 nm Au nanoparticles with gaps of 3.3 ± 1 nm on a DNA origami platform. The innovative structure ensures ultra-high EF between nanoparticles without occupying the gaps between them. Further, a series of functional complicated nanostructures were assembled on DNA origami platform, including heterodimers [53], nanostar dimers [54], bowties [55], nanocavities [56], nanoforks [57] (Figure 2d), etc.

Tip-enhanced Raman spectroscopy (TERS) [58,59,60], an extension of SERS, enhances the Raman signal of the analyte on the substrate by a plasmonic tip. The apex of a tip with a high aspect ratio induces the lighting rod effect and localizes the electromagnetic field into a tightly confined space [61]. With a sophisticated mechanical system, precise coupling of the tip and plasmonic substrate enables much larger EF, similar to the NPoM. Zhang et al. [14] realized single-molecule TERS in an ultra-high vacuum and low temperature (Figure 2e). The plasmonic tip and substrate form a nanocavity. By spectrally matching the SPR of the nanocavity and the electronic transition of the molecule, both Raman excitation and emission are enhanced. The chemical mapping of a single molecule with subnanometer spatial resolution was achieved. Furthermore, nonlinear TERS pushed the spatial resolution down to 0.5 nm [62]. Two adjacent molecules of very similar structure with van der Waals contact can be distinguished. Although single-molecule TERS is typically implemented at cryogenic temperature, recently, several reports have shown that TERS can achieve single-molecule sensitivity at room temperature [48]. Park et al. [63] performed variable temperature (90–300 K) single-molecule TERS of malachite green. From temperature-dependent line narrowing and splitting, the ultrafast vibrational dephasing, conformational heterogeneity, and intramolecular coupling were quantified. Liu et al. [64] proposed fishing-mode TERS (FM-TERS) to study single-molecule junctions in different conduction states (Figure 2f). A bias voltage is applied between the tip and substrate to tune the conduction states of the single-molecule junction. Single-molecule conductance and Raman spectrum are simultaneously acquired at room temperature by FM-TERS.

Limited by the optical diffraction limit, the focal spot of the excitation beam is much larger than the hotspot. Improving coupling efficiency from far-field to near-field is a complementary approach to increase EF. Ahmed et al. [65] proposed a nanoantenna for directional SM-SERS. Wang et al. [66] fabricated a nanoantenna chip containing more than 10^3^ nanoantennas with top-down fabrication methods (Figure 2g). Each nanoantenna consists of a Ag dimer with a 5 nm gap and a Ag ring, all on a SiO_2_ spacer layer on a Ag mirror. The Ag mirror supports the SPR excited on the dimer, and the Ag ring converges the surface plasmon polaritons (SPPs) on the mirror to the dimer. The Ag mirror and Ag ring provide an extra two orders of magnitude EF. Chen et al. [15] demonstrated a plasmonic device consisting of a sub-10 nm wide and 1 μm long nanoslit, a cavity, and two Bragg-mirror gratings for single-molecule nucleobase sensing (Figure 2h). The cavity efficiently couples the excitation light into SPPs and guides them into the nanoslit. Two Bragg-mirror gratings reflect SPPs back into the slit-cavity to further strengthen the enhanced field. In TERS, a spiral tip fabricated by metal deposition and direct laser writing is proposed [67,68]. Through the spiral design, the mirror symmetry of the plasmonic tip is broken, avoiding destructive interference of the coupled SPPs, achieving 30% greater EM enhancement than the conical tip.

Reproducible SM-SERS experiments and data rely on the plasmonic nanogap with precise size control. So far, fabricating such a precise nanostructure is still a challenge in a variety of environments.

### 3.2. Strategies for Improving the Probability of Molecules in Hotspots

As mentioned above, in SM-SERS experiments based on random adsorption, it is a rare event that molecules are located in hotspots due to ultra-low concentration and narrow hotspots with sparse distribution. Long observation time is required for statistical confidence, suffering from free diffusion of molecules. An approach is to chemically bind the molecule into the gap. In TERS, the hotspot generated by the tip is scanned along the substrate with scanning probe microscopy (SPM). All of the analytes on the substrate are present in the hotspot in one scan. However, single-molecule TERS is still limited by environment and imaging speed.

**Figure 3:**
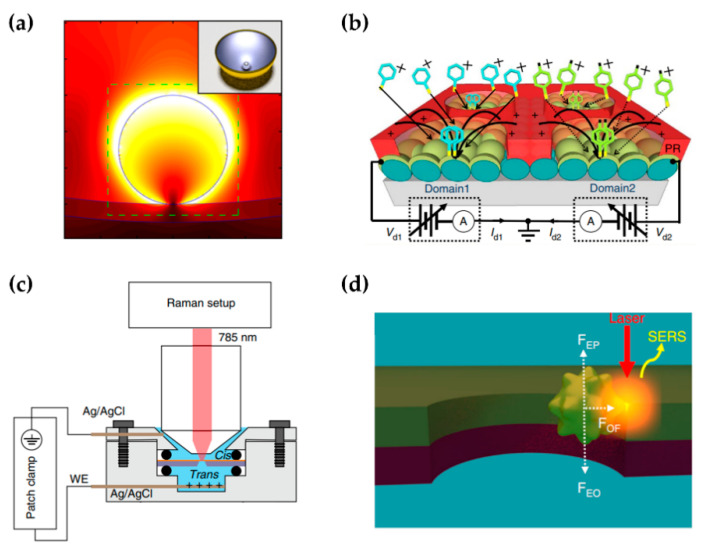
Strategies to improve the probability of molecules in hotspots. (**a**) FDTD simulation for a Ag nanoparticle on a warped Au substrate. Figure reproduced from [72]. Inset: the schematic of the NPoM with warped substrate. FDTD, finite difference time domain. (**b**) The SERS substrate with programmable localized electrodynamic precipitation. Figure reproduced from [73]. PR, patterned photoresist layer. (**c**) Schematic of the setup for nanoslit SERS. Figure reproduced from [15]. WE, working electrode. (**d**) Electro–plasmonic trapping due to the balance between the electrophoretic (F_EP_), electroosmotic (F_EO_), and plasmonic gradient force (F_OF_). Figure reproduced from [74].

Expanding the volume of hotspots directly improves the probability of molecules in hotspots. Langmuir–Blodgett assembly is a powerful technique for assembling a large-area monolayer of anisotropic blocks [69]. Tao et al. [70] assembled Langmuir–Blodgett monolayers of aligned Ag nanowires covering over 20 cm^2^ as a SERS substrate. Zhang et al. [71] reported a wrinkled, nanoporous Au film containing a high density of hotspots with EF of 10^9^. Mao et al. [72] designed a warped substrate instead of a flat substrate for NPoM by transformation optics, resulting in broadband enhancement in both magnitude and volume (Figure 3a). Single-molecule detection with only 60 s soaking time was achieved.

Another approach is to deliver molecules to the hotspots instead of random adsorption. Lin et al. [73] proposed a SERS substrate with programmable localized electrodynamic precipitation (Figure 3b). By applying a bias voltage to the substrate, analytes as far as centimeters away can be directed to the hotspots. Chen et al. [15] separated the electrolyte solution into two chambers by a nanoslit chip (Figure 3c). By applying a positive bias voltage, the nanoslit provides the only path from the cis to the trans chamber. Adsorption and translocation of molecules is manipulated by modulating the transmembrane voltage to match the slow sampling speed of SERS. Huang et al. [74] trapped the analyte molecules adsorbed on Au nanoparticles to the sidewall of a nanopore for minutes through a combination of electroosmotic force, electrophoretic force, and plasmonic gradient force (Figure 3d). In addition, localized optical heating, plasmonic optical trapping, and magnetic wells have been used for single-molecule manipulation in nanopore-based systems [75,76,77].

### 3.3. Nonmetallic and Hybrid Substrate

Conventional SERS substrates are dominated by noble metals, such as Au and Ag. However, Au and Ag typically suffer from high cost, heterogeneity, lack of stability, and bio-incompatibility. Nonmetallic materials, such as semiconductors, insulators, organic materials and 2D materials [78,79], can serve as alternatives or as complements to noble metal substrates. A range of nonmetallic SERS substrates has been proposed based on different principles, including total internal reflection [80], interference [81], optical amplification [82], Mie resonance [83], charge-transfer resonance [84], etc. CM is a common strategy for nonmetallic SERS substrates. However, CM-induced enhancement is relatively low; thus reports of SM-SERS with nonmetallic substrate are relatively rare.

Cong et al. [85] reported a sea urchin-like W_18_O_49_ nanowire as a SERS substrate, and realized a detection limit concentration of 10^−7^ M and EF of 3.4 × 10^5^. The oxygen deficiencies strengthen the substrate–analyte interactions and enable significant Raman enhancement. Keshavarz et al. [86] proposed a Si@SiO_2_ quantum probe as a SERS probe for biomolecular and intracellular sensing due to its chemical stability and biocompatibility. Superior EF of 10^7^ at pM concentration was achieved. On the other hand, a combination of metal and nonmetal has also been adopted for SM-SERS. CM-induced enhancement can relax requirement on EM-induced enhancement. He et al. [87] coupled Ag nanoparticles with Si nanowires to achieve an EF of 10^10^. Shell-isolated nanoparticle-enhanced Raman spectroscopy (SHINERS) [88], coating Au nanoparticles with ultra-thin and pinhole-free Si shells, eliminated the influence of adsorbing analytes and has been widely used to investigate chemical reactions. In summary, it is still a challenge to develop nonmetallic substrates with single-molecule sensitivity to extend SM-SERS to more applications.

## 4. Application of SM-SERS

### 4.1. In Situ Monitoring of Catalytic Reactions

The rational design of highly efficient catalysts depends on understanding the structure–activity relationship and reaction mechanism at the single-molecule level. SM-SERS, with its ultra-high surface sensitivity, is a powerful tool for in situ monitoring and elucidating the mechanisms of catalytic reactions [89,90,91,92].

**Figure 4:**
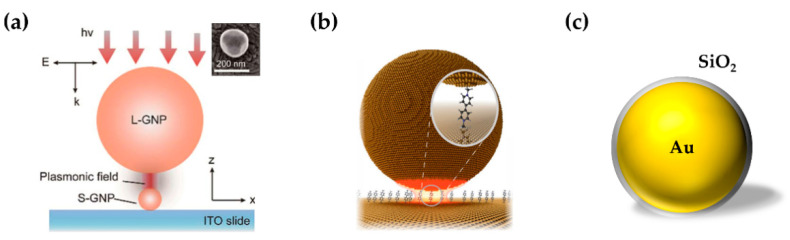
Monitoring of catalytic reactions by SM-SERS. (**a**) The Au dimers, consisting of two nanoparticles sized 200 nm and 13 nm. Figure reproduced from [95]. Inset: an SEM image of the Au dimers. L-GNP, large gold nanoparticles. S-GNP, small gold nanoparticles. ITO, indium tin oxide. (**b**) Self-assembled 80 nm NPoM for eliciting EF for SM-SERS. Figure reproduced from [96]. (**c**) SHINERS, Au core SiO_2_ shell nanoparticles.

Noble metal nanoparticles can act as catalysts on which plasmon-induced hot electrons are transferred to reactants [93]. Zhang et al. [94] discovered a new plasmon catalyzed reaction of p-nitrothiophenol (pNTP) by SM-SERS. In previous studies, dimerization of pNTP to 4,4-dimercaptoazobenzene (DMAB) was thought to occur on the surface of Au nanoparticles. Nevertheless, dissociation of pNTP to thiophenol (TP) was observed at an ultra-low concentration (10^−9^ M), and plasmon-induced hot electrons provided the activation energy for the dissociation. Zhang et al. [95] reported direct SERS tracking of the catalytic reactions at a 13 nm Au nanoparticle. Spatially isolated Au dimers, consisting of two nanoparticles sized 200 nm and 13 nm, integrate catalytic activity and SERS into a single entity (Figure 4a). Further, combining SM-SERS with electrochemistry could drive the reactions by applying the bias voltage to inject or extract electrons from the analytes [64]. Nijs et al. [96] studied hot-electron reduction and oxidation dynamically with a NPoM configuration by SM-SERS (Figure 4b).

Generally, the catalytic activity of noble metals is relatively low. To expand SERS to a catalytically active transition metal, Tian et al. [97] employed Au nanoparticles coated with ultra-thin shells of a transition metal such as Pt, Pd, Ni, or Co. In such a scheme, the Raman signal of analytes adsorbed on the transition metal shell is enhanced by the Au core. SHINERS [88], already mentioned in the previous section, improve the chemical and thermal stability of the noble metal core, enabling in situ monitoring of catalytic reactions in harsh conditions (Figure 4c).

### 4.2. Characterization of Molecular Nanoelectronics

Molecular nanoelectronics use single molecules as building blocks of logic units, electronic devices, and circuits, with small size, low power consumption, and unconventional properties [98]. However, poor reproducibility limits the application of molecular nanoelectronics. The molecular structure in nanoelectronic devices is not clear by conventional conductance measurements [99]. SM-SERS, as a complementary characterization method, could study molecular conformation and metal–molecule interface structure and charge transfer at room temperature.

**Figure 5:**
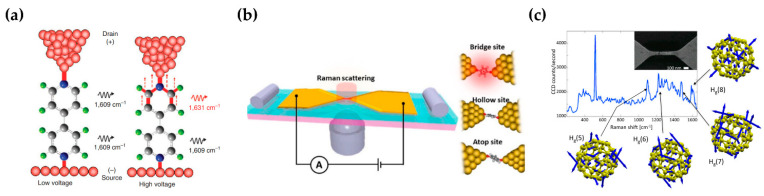
Characterization of molecular nanoelectronics by SM-SERS. (**a**) The schematic of the Au–4bipy–Au junction with low/high bias voltage. Figure reproduced from [64]. (**b**) Simultaneous SERS and conductance measurements for single-molecule junction. Figure reproduced from [100]. Right: three metastable states in BDT junctions. (**c**) SERS of C_60_ in an electromigrated junction. Figure reproduced from [102]. Inset: an SEM image of an electromigrated junction.

Liu et al. [64] observed voltage-dependent peak splitting due to the different bonding interactions occurring on the Au–4bipy–Au junction (4,4′-bipyridine, 4bipy) by FM-TERS (Figure 5a). Kaneko et al. [100] studied the selectivity of adsorption sites in a single-molecule junction by simultaneous SERS and conductance measurements (Figure 5b). The conductance response revealed the coexistence of three metastable states in 1,4-benzenedithiol (BDT) junctions. The SERS results showed selectivity of adsorption site toward “bridge sites”. Han et al. [101] studied the influence of SPR on interfacial charge transfer in TiO_2_–MBA–Au assemblies (MBA, mercaptobenzoic acid) by SERS. Li et al. [102] studied voltage tuning of vibrational mode in C_60_ junctions (Figure 5c). Applying a bias to the junction, vibrational mode softening approximately quadratic in the bias voltage occurs. This stark effect alone cannot explain the phenomenon, but alteration of the molecular charge state is responsible for the effect.

### 4.3. Single-Molecule Sensing

Single-molecule sensing is an important subject in biochemical analysis. In single-molecule sensing, observation of each molecule is an independent event, revealing information that is drowned out by statistical average, such as sample heterogeneity, molecular mechanism, and complex kinetic rate [8]. For fluorescence or conductance measurements, one-dimensional data provide limited information that different molecules may yield similar signals. SM-SERS provides rich spectral information conducive to distinguishing a variety of similar analytes [75,103,104], which is of great significance in DNA sequencing, protein identification, etc. Further, Zhou et al. [105] utilized the rich spectral information to probe the orientation and oxygenation of single molecules.

**Figure 6:**
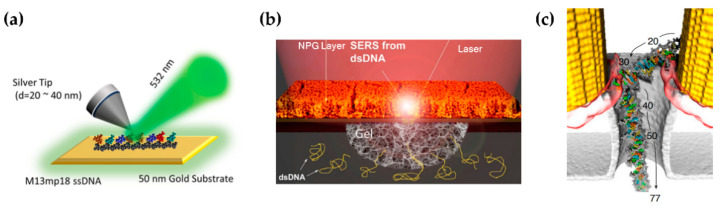
Biochemical sensing by SM-SERS. (**a**) Identification of single-stranded DNA by single-molecule TERS. Figure reproduced from [107]. The DNA molecules are deposited on the Au substrate. (**b**) The schematic of the plasmonic nanopore in the nanoporous film. NPG, nanoporous gold. Figure reproduced from [109]. (**c**) DNA sequence trapped in plasmonic nanopore. The base pairs are numbered in ascending order from tail to front of the molecule. Figure reproduced from [110].

TERS can achieve both single-molecule sensitivity and subnanometer spatial resolution. After depositing the analytes on the substrate, the chemical structure of single molecules can be imaged [14,62]. Viruses [106], single-stranded DNA [107], and RNA [108] have been identified by single-molecule TERS (Figure 6a).

A plasmonic nanopore is a single-molecule sensor widely used in solution at room temperature. The electrolyte solution is divided into two chambers by an electrically insulating membrane on which the nanopore is a nanoscale aperture. A bias voltage applied across the membrane drives the charged analyte through the nanopore [111]. The ionic current and SERS signal are detected when the analytes pass through the nanopore, which can be used for single-molecule sensing. Translocation of amino acids, nucleobases, and DNA has been probed through plasmonic nanopores [112,113]. Nevertheless, in SERS measurement based on nanopores, translocation time is on the order of microseconds, which is insufficient for the spectrum acquisition on the order of milliseconds. Zong et al. [114] utilized dynamic SERS to improve the signal-to-noise ratio (SNR) even when the acquisition time cannot catch up with the diffusion time of molecules. With insufficient acquisition time, SERS was extracted via data processing from time-dependent spectral series. Hubarevich et al. [109] fabricated a plasmonic nanopore in a thick nanoporous film and added hydrogel at the bottom of the nanoporous membrane, which significantly reduced the velocity of translocation (Figure 6b). Single-molecule trapping has been adopted to prolong the residence time of molecules within nanopores [15,74,75,76,77], as described in Section 3.2. Although a series of single molecules have been identified in nanopore by SM-SERS, more delicate operations, such as stepwise displacement of DNA strands through the nanopore, have not been realized in experiments (Figure 6c) [110].

## 5. Several Challenges in SM-SERS

As mentioned in the previous sections, with vigorous development in recent years, SM-SERS has achieved great success in various fields. However, SM-SERS has not been fully developed yet. Several challenges still need to be overcome to further boost advancement.

First, although EM and CM have explained a lot of SERS phenomena in the past two decades, some experimental events are still confusing. Indeed, the mechanism of SERS is not completely clear, especially for single-molecule events. It has been proposed that some previously ignored physical effects such as Rayleigh scattering, discrete interaction, and quantum interaction deserve to be considered in the mechanism [23,24,25,26]. Thus, it is necessary to extend the SERS theory to take physical effects into account. Further, the extended SERS theory can also provide insights into the design of advanced SERS systems.

Second, the intrinsic fluctuation of SM-SERS disturbs the data analysis algorithm, but on the other hand also implies single-molecule information. An algorithm for extracting single-molecule chemical information from SM-SERS data has not been developed. The study of spectral fluctuation and blinking could deepen the understanding of SM-SERS data and develop advanced data analysis algorithm for SM-SERS.

Third, in airborne environments and solutions at room temperature, the reproducibility of SM-SERS experiments is still poor, which is attributed to two aspects. On the one hand, the Brownian motion of molecules in the hotspot induces spectral fluctuations. Delicate single-molecule manipulation techniques are required to localize molecules to the same position of the hotspot or even drive molecules stepwise through the hotspot [110,115,116]. On the other hand, fabrication of SM-SERS substrates is also important to obtain high-reproducibility substrates.

Fourth, nonmetallic substrates greatly broaden the application of SERS. However, due to the poor SERS activity of nonmetal, nonmetallic substrates with single-molecule sensitivity have rarely been reported. Material structural and hotspot engineering may lead to the development of SM-SERS with nonmetallic substrates [78].

Fifth, ultrafast SERS, incorporating SERS with an ultrafast technique, has opened a new direction due to its fs and ps time resolution and higher sensitivity [49,117]. Ultrafast SERS can provide real-time monitoring of molecular structure and bond breaking and forming, enabling elucidation of mechanisms of plasmon-induced photochemical and photophysical processes.

## 6. Conclusions

In this review, we first elucidate the key concepts of SM-SERS, including EF, spectral fluctuation, and experimental evidence of single-molecule events. Next, we systematically discuss advanced implementations of SM-SERS, including substrates with ultra-high EF and reproducibility, strategies to improve the probability of molecules in hotspots, and nonmetallic and hybrid substrates. Then, several examples for the application of SM-SERS are proposed, including catalysis, nanoelectronics, and sensing. Finally, we summarize the challenges and future of SM-SERS. We hope this literature review will inspire the interest of researchers in more fields. SM-SERS is expected to make more progress in science and application in the near future.

## Data Availability

Not applicable.
